# Evidence of dietary protein restriction regulating pupation height, development time and lifespan in *Drosophila melanogaster*

**DOI:** 10.1242/bio.042952

**Published:** 2019-06-15

**Authors:** Sudhakar Krittika, Alisha Lenka, Pankaj Yadav

**Affiliations:** 1 Fly Laboratory # 210, Anusandhan Kendra-II, School of Chemical and Biotechnology, SASTRA Deemed to be University, Thanjavur 613401, Tamil Nadu, India; 2 Gautam Buddha University, School of Biotechnology, Yamuna Expressway, Near, PariChowk, Greater Noida, Uttar Pradesh 201308, India

**Keywords:** *Drosophila melanogaster*, Diet restriction, Pupation height, Development time, Lifespan

## Abstract

Fitness and behavioral traits are optimized according to the rearing environment to ensure survival of most organisms including fruit flies *Drosophila melanogaster*. Fruit flies are known to uphold various trade-offs in their lifespan, development time, fecundity, etc., to confer better survival in the particular exposed environment. The diet of *D. melanogaster* plays a major role between larval and adult fitness or fitness related traits; its role in the regulation of correlations between pupation height, pre-adult development and adult fitness has not been studied empirically. In our study, we assayed the effect of restricting dietary protein alone from the larval stage to adult stage in fruit flies and studied development time, pre-adult survivorship, pupation height, larval feeding rate and their corresponding lifespan under a light/dark cycle (LD12:12 h). We found that under very low protein concentration in diet, development time and lifespan of the flies increased significantly, along with decreased pupation height and vice versa, while pre-adult survivorship remained unchanged across diets. The results from our study can be taken to suggest that development time is negatively and positively correlated with pupation height and adult lifespan respectively. Thus, a higher protein restriction decreases pupation height and increases development time and vice versa, thereby emphasizing differential alterations taken up by various fitness traits, probably to enhance the overall organismal fitness.

This article has an associated First Person interview with the first author of the paper.

## Abstract (precis)

**Summary:** In studying protein-restricted diet-mediated interplay between major traits during pre-adult to adult stages in *Drosophila melanogaster*, we found various existing positive and negative correlations being challenged and supported upon differing diets.

## INTRODUCTION

The approach of limiting one or more nutrient intake below *ad libitum* (dietary restriction; DR henceforth) is a commonly used manipulation in diet to study various fitness traits, physiology, metabolic and nutrient sensing pathways in various model organisms (Ormerod et al., 2017; Stefana et al., 2017). Addressing DR effect on life-history traits, development and molecular mechanisms has played a vital role in understanding the importance of the restricted nutrient/s in *Drosophila melanogaster* (Mair et al., 2003, 2005; Bruce et al., 2013) due to their shorter lifespan, easier maintenance protocols and well exploited genetics. Though extensive studies have been reported on the effect of DR on adults, very little is known about the impact of DR at the larval stage of fruit flies. Juvenile nutrition approaches demonstrating that yeast deprived larvae showed increased development time, smaller body size and increased lifespan (Tu and Tatar, 2003; Stefana et al., 2017), with no significant difference in adult mortality as compared to the larvae that were fed a restricted or normal diet (Tu and Tatar, 2003) were reported. Although nutritional conditions of larvae can affect the subsequent body size and fecundity of adults, these are not sufficient to have an effect on lifespan (Tu and Tatar, 2003). Contrarily, studies have shown that larval competition for food (Klepsatel et al., 2018) and nutrient availability during adulthood can potentially impact the lifespan of *D. melanogaster* in nature. Fitness traits such as pre-adult survivorship, development and lifespan are affected not only by diet, but also by oviposition site (Yang et al., 2008; Lihoreau et al., 2016; Silva-Soares et al., 2017), pupation height (a behavioral trait; Chiang and Hodson, 1950; Sameoto and Miller, 1968) and environmental factors including stress due to starvation and low temperature (Nunney and Cheung, 1997; Hoffmann et al., 2005; Aggarwal et al., 2013). While multiple studies have exhaustingly focused on the role of diets on lifespan, development and fecundity (Metaxakis and Partridge, 2013; Lee, 2015; Ormerod et al., 2017), we aimed to study lifespan, development and pupation height differences in the same flies using different protein restricted diets from the pre-adult stage to death, in order to obtain a concrete correlation between these traits.

In *D. melanogaster*, trade-offs in life-history traits such as pre-adult development time, lifespan and reproduction with preference for ingestion of proteins, amino acids and/or carbohydrates have been extensively studied (Lee et al., 2008; Grandison et al., 2009; Lee, 2015). Fruit flies are known to make complex foraging choices under DR, especially when female flies have to decide between their own nutrition or that of their offspring (Lihoreau et al., 2016), while nutrient-restricted mother flies are capable of transferring the plasticity of survival in a nutrient-restricted environment to their offspring as well (Crofton et al., 2018). The trade-offs in fruit flies are highly flexible with environmental conditions and are strongly influenced by nutrition, larval crowding (Klepsatel et al., 2018) and stress conditions (Nunney and Cheung, 1997; Hoffmann et al., 2005; Aggarwal et al., 2013). Organisms tend to forage on multiple food sources to ensure their nutritional satiety (Simpson et al., 2004). In fruit flies, when the larvae are deprived of nutrient availability, the juveniles have to make important foraging decisions and trade-offs that will ensure their fitness. With more focused research being done on the effect of diet on development and lifespan, the less explored trait in *D. melanogaster* is its choice of pupating height. When larvae pupate higher up, it increases their chance of successful eclosion, and hence might reflect enhanced fitness in normal populations of *D. melanogaster* (Buck et al., 2000). But the choice of pupation height is influenced as a combined response to selection (Joshi and Mueller, 1996; Chippindale et al., 1997; Prasad et al., 2001), apart from several other factors such as texture of food, temperature (Vandal and Shivanna, 2007), light (Paranjpe et al., 2004) and larval density (Sokal et al., 1960; Joshi and Mueller, 1993). Hence, assessing the decisions of larvae regarding their development time and pupation height with respect to the availability of diets will be helpful to study its long term effects, especially on lifespan.

Our present study aimed to examine the effect of reduced protein diets on development time, pre-adult survivorship, larval feeding rate, pupation height and adult lifespan, and thereby assess the influence of diet on pre-adult and adult fitness traits in *D. melanogaster*. The results revealed that protein concentration in the diet is inversely related to development time and adult lifespan, while pre-adult survivorship remained unaffected. Diet also variably influences pupation height and larval feeding rate, wherein very low protein concentration is associated with lower pupation height and reduced larval feeding rate, while restricting protein by half that in the control food is capable of achieving pupation height equal to that of the control. Although previous studies show a positive as well as negative correlation between pre-adult development time and pupation height (Buck et al., 2000; Casares and Carracedo, 1987 respectively), the effect of DR on this correlation still remains unclear. Therefore, our study aimed to assess the influence of diet on these two traits alongside deciphering their correct correlation status. Further, our study tested the lifespan of flies under different DR using the same flies that have eclosed from the pupation height, pupation time, pigmentation time and development time assays, thereby enabling comparison of pre-adult and adult life-history traits relationship in the same flies. Thus, our study reports a combined assessment of various fitness traits, while the influence of diet on pupation height and larval feeding rate in *D. melanogaster* reported here is the first ever to our knowledge.

## RESULTS

### Shortening of pupation time on DR

**Fig. 1.**
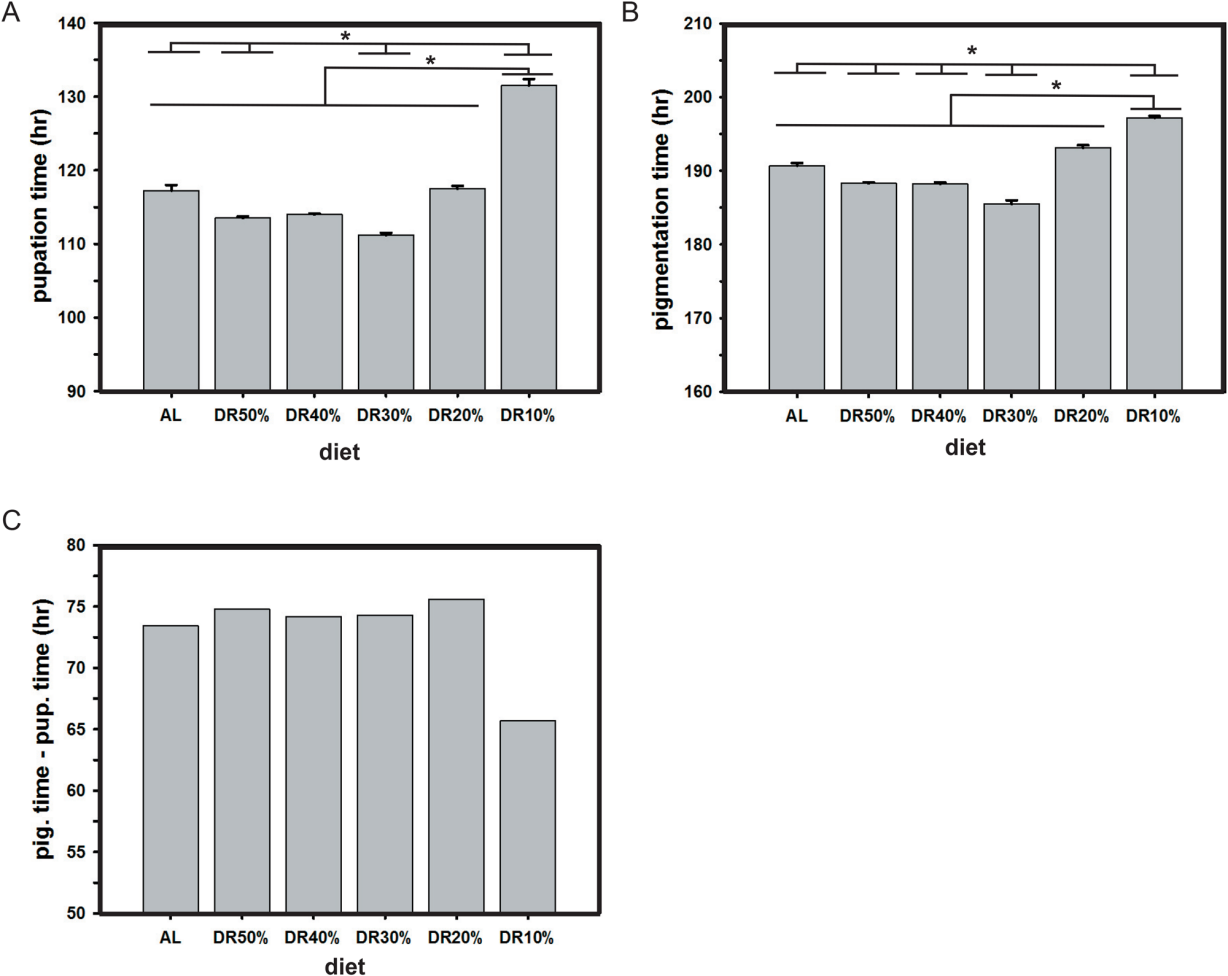
**Dietary restriction (DR of protein) shortens pupation and pigmentation time.** Upon imposed DR, flies show varied pupation time (A), pigmentation time (B), diet based pigmentation time and pupation time difference (pig. time-pup. time) (C). DR10% shows higher pupation and pigmentation time compared to AL and other DRs and the statistically significant differences are represented by horizontal lines above the bars. A total of five vials (30 eggs per vial) were used under different DR. The error bars are standard error around the mean (s.e.m.) and the asterisks indicate statistical significance (*P*<0.05).

To study the effect of varied yeast (protein) concentration alone on pupation, we assayed their durations in fruit flies. ANOVA followed by post hoc multiple comparisons using Tukey's test revealed that the pupation time under various diets (*F*_5,24_=94.05, *P<*0.0001; Fig. 1A, Table 2) was significantly different. The effect of DR10% was significantly higher as compared to that of control (AL) and other DRs; while AL was not significantly different from DR20% and DR40%; similarly DR30% was not different from DR40% and DR50%. Thus, there is a significant effect of diet protein restrictions on pupation time in fruit flies.

### Shortening of pigmentation time in DR

The pigmentation time taken by the flies is seen to vary upon altered diet protein concentrations alone. ANOVA followed by post hoc multiple comparisons using Tukey's test revealed that the pigmentation time is significantly altered in DR as compared to that of AL food (*F*_5,24_=51.7, *P<*0.0001; Fig. 1B, Table 2). Similar to pupation time, DR10% shows significantly higher pigmentation time as compared to that under AL and other diets. The results also show that AL and DR20% are not significantly different, while DR40% and DR50% are not different between themselves. It is also observed that the time difference between the pigmentation and pupation across diets is almost equal except for DR10%, where it shows reduced time difference between the pupation and pigmentation (Fig. 1C). These results suggest that the pigmentation time in flies is significantly different based on restricted diet protein.

### Differential egg-to-adult development duration under varied yeast concentrations

**Fig. 2.**
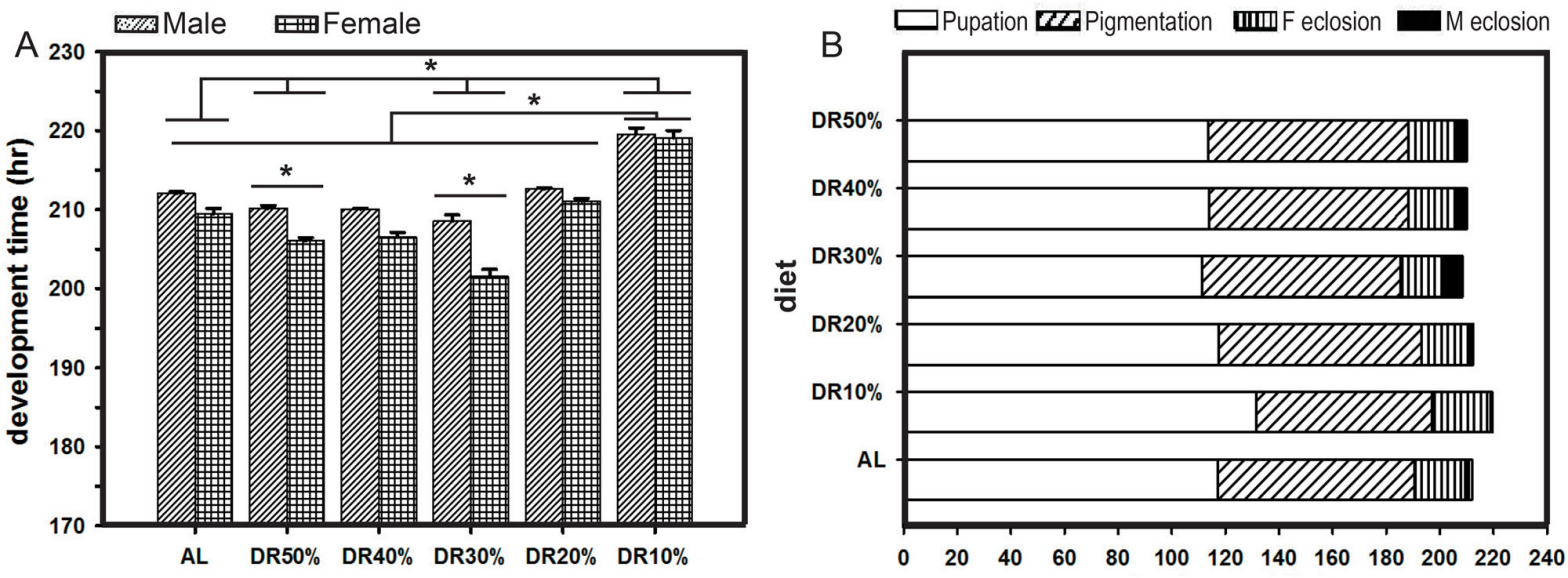
**Varied protein reduction causes differential egg-to-adult development duration.** Low protein levels lengthen average pre-adult development time of fruit flies, thereby showing significant effect of diet (A). Role of protein restriction in the duration of various pre-adult stages (B) is multifarious under different diets. The overall development time is longer at DR10% in both males and females. All other details are same as in Fig. 1.

We assayed the effect of restricting protein concentration on three different stages of the pre-adult development in fruit flies. The results showed that the pre-adult development time under DR10% was drastically lengthened (Fig. 2A). ANOVA on the development time data showed a statistically significant effect of diet (*F*_5,48_=67.8, *P<*0.0001), sex (*F*_1,48_=42.9, *P<*0.0001) and diet×sex (*F*_5,48_=3.7, *P<*0.0064; Table 2). Post hoc multiple comparisons using Tukey's HSD test revealed that the flies show significantly increased development time at the lowest protein concentration of DR10%, and decreased development time in DR30% and DR50% as compared to that of AL, while the sex-based effect is seen only in DR30% and DR50%, where the development time of males is significantly higher than that of females. Similarly, when the factors of sex and diet are taken into account simultaneously, DR30% females develop faster as compared to all the other males and females across the diets. Thus, these results suggest that the altered development time of flies is a response to their dietary protein requirement and the sex of the flies, thereby showing differential development time differences (Fig. 2A,B) to compensate for their nutritional needs.

### Pre-adult survivorship and larval feeding rate under different DR

**Fig. 3.**
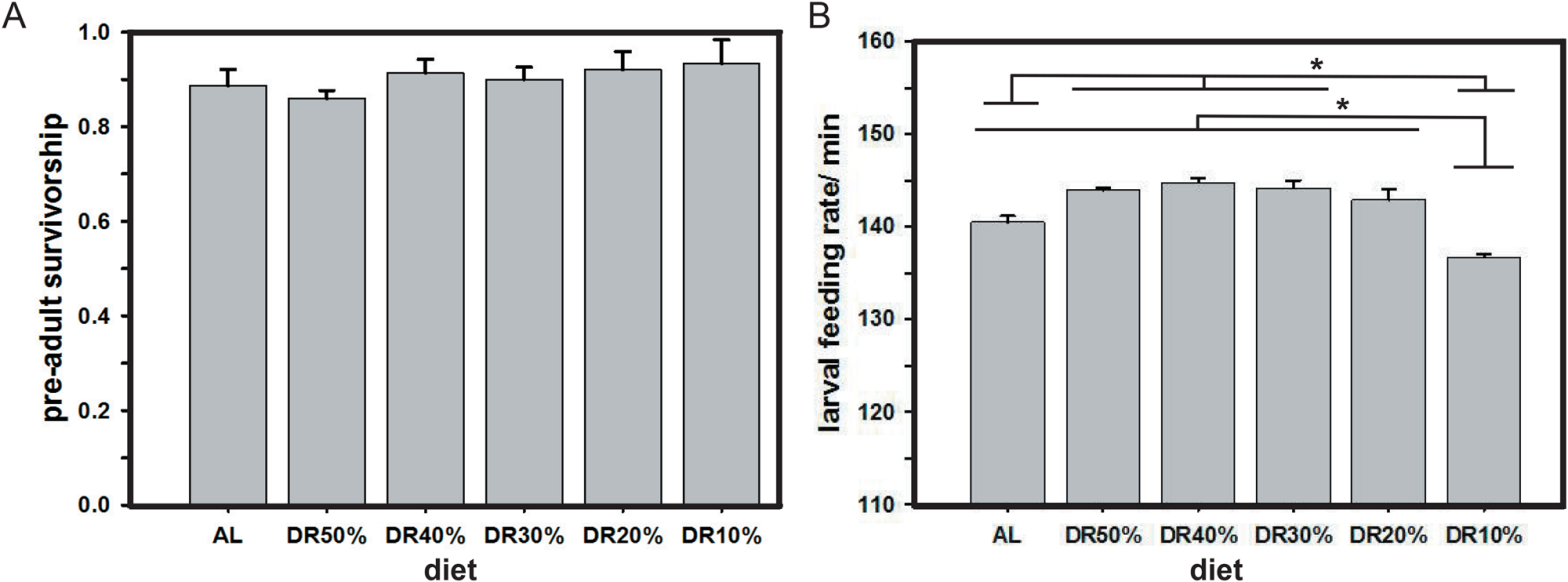
**Reduced diet protein does not affect pre-adult survivorship, but influences larval feeding rate.** Different range of restricted protein in the diet does not affect the mean pre-adult survivorship of fruit flies (A), while the larval feeding rate at DR10% is the slowest as compared to AL and other DRs (B). All other details are same as in Fig. 1.

The pre-adult survivorship under different diets was found to be unaffected (Fig. 3A), and ANOVA revealed no significant effect of diet (*F*_5,24_=0.59, *P<*0.7096), suggesting that varying diet concentrations does not affect pre-adult survivorship. While the larval feeding rate showed significant effect of diet (*F*_5,24_=19.49, *P<*0.0001), wherein the larvae feeding rate is lowest at DR10% as compared to that of AL and other DRs. In addition to this, larval feeding rate in AL is not significantly different to that in DR20% alone, thereby indicating possibilities of similar nutritional requirements between them.

### Pupation height under different DR

**Fig. 4.**
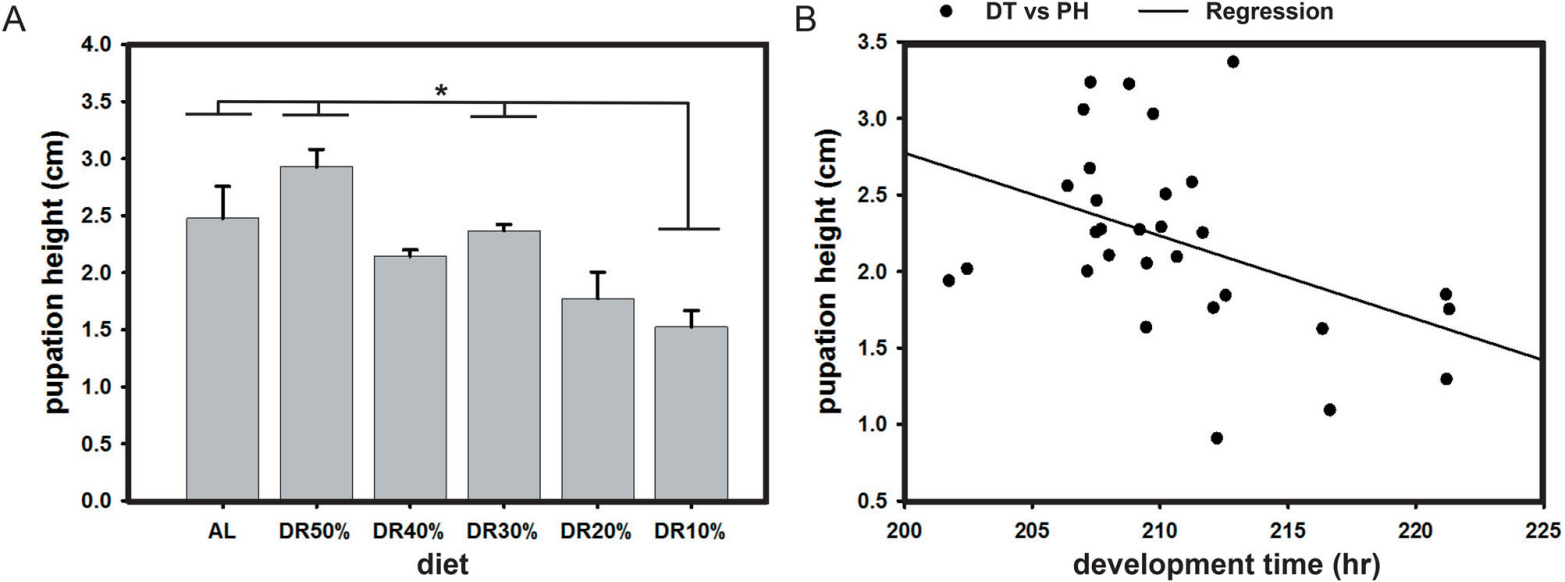
**Restricted diet protein influences pupation height.** Restricted diet protein affects pupation height in fruit flies. The pupation height under DR10% is significantly lower as compared to AL, DR30% and DR50% (A). A negative correlation (*r*=−0.43; *P*=0.01) exists between development time (DT) and pupation height (PH) (B). All other details are same as in Fig. 1.

We assayed the effect of different protein restricted diets on the pupation height of flies. ANOVA on the pupation height data showed a statistically significant effect of diet (*F*_5,24_=6.93, *P*=0.0004; Table 2). The results show a significant difference in pupation height across AL and different diets, wherein the pupation height in DR10% is significantly lower compared to that under AL, DR30% and DR50% (Fig. 4A). Post hoc multiple comparisons using Tukey's HSD test on pupation height data shows in different diets the pupation height of flies is significantly lower at DR10% than AL, while under other diets it is not statistically different from AL. These results suggest that there exists a significant difference in pupation height of flies under varied protein restricted diets as compared to AL, alongside a moderate negative correlation (*r*=−0.43; *P*=0.01) existing between development time and pupation height (Fig. 4B).

### Delayed development causes increased adult lifespan under low protein diet

**Fig. 5.**
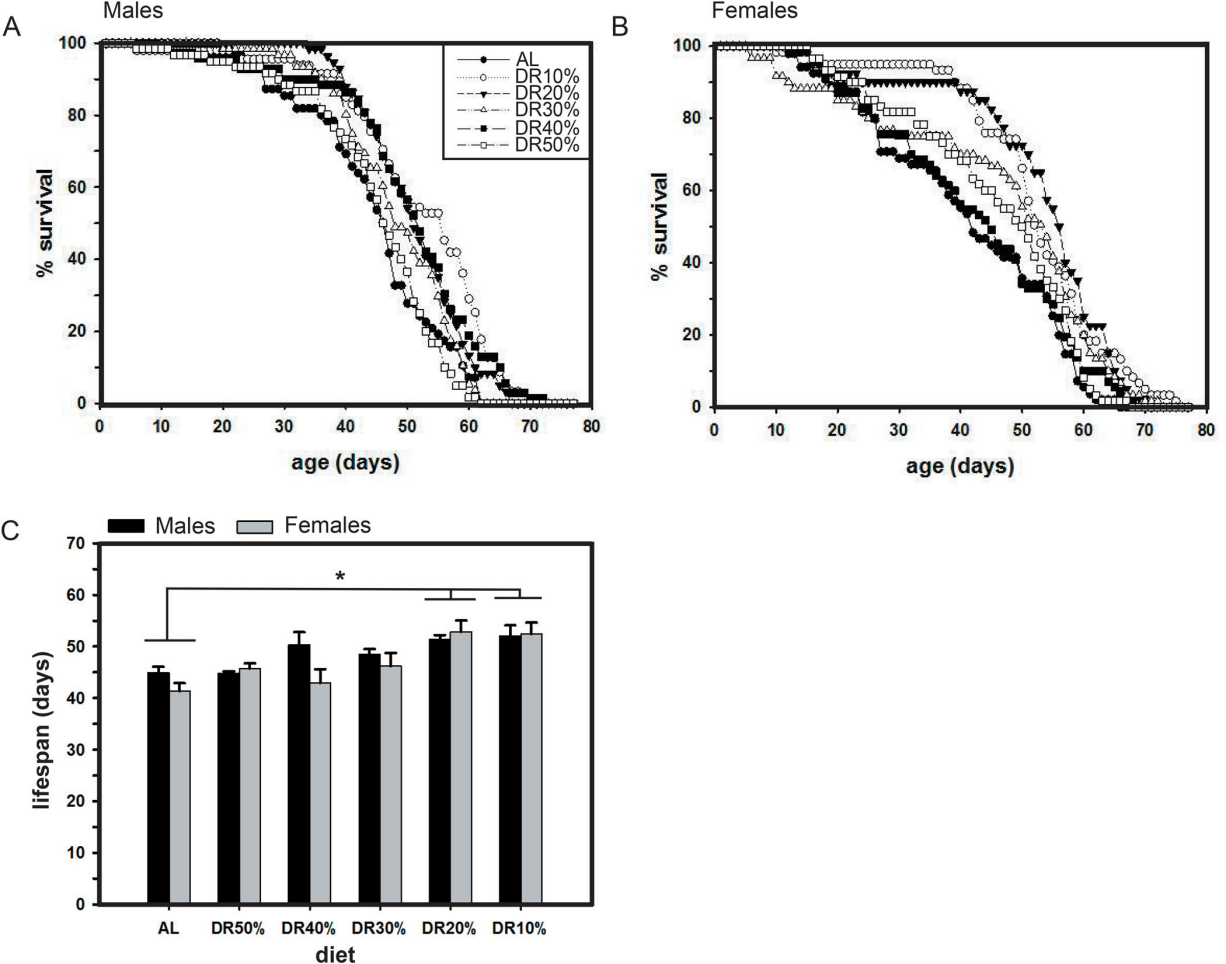
**Effect of DR on lifespan of fruit flies.** DR causes lifespan variations in males (A) and females (B) at restricted protein concentrations as low as DR10% and 20%, while there exists no significant sex based differences. The average lifespan of the flies across different diets shows extension under DR10% (C). All other details are same as in Fig. 1.

ANOVA revealed a statistically significant effect of diet (*F*_5,60_=4.5, *P*=0.0014), but not sex (*F*_1,60_=1.5, *P*=0.2259; Table 2). Post hoc multiple comparisons using Tukey's test revealed that the difference in lifespan under DR is significantly higher under DR10% and DR20% than in AL (Table 2, Fig. 5A,B). At DR10%, the development time and lifespan was found to be increased, while this was not evident in DR20%. At DR20%, lifespan was higher than in AL even though no difference existed in development time as compared to AL, showing that DR20% of protein was capable of increasing lifespan (adult trait), but was not sufficient to significantly alter development time (pre-adult trait). These results show that even under the lowest protein concentration, an increase in development time and lifespan can be witnessed where the influence of DR is equivalent for both males and females. Thus, the results from our study suggest that across different diets pre-adult development time and adult lifespan are positively correlated in fruit flies (Fig. 5C).

## DISCUSSION

The nutritional intake of *D. melanogaster* has been variably altered by various mechanisms like calorie restriction, protein:carbohydrate ratios and DR (Rogina and Helfand, 2004; Bruce et al., 2013; reviewed in Kapahi et al., 2017). An array of research on DR via protein restriction alone has gained pace in *D. melanogaster*, aimed at studying the role of reduced protein intake in the regulation of pre-adult and adult nutrition requirements, development time and lifespan (Rodrigues et al., 2015; Klepsatel et al., 2018). However, most of these studies comprise assaying one or a few traits together and use flies from different groups in different experiments even if they are studied in the same laboratory, thereby creating differential response between flies even if they belong to the same strain. We sought to understand how dietary yeast can influence the development time, pre-adult survivorship, pupation height and their corresponding lifespan of *D. melanogaster*, by using the same set of flies to assess all the parameters from development time to lifespan. This enables us to understand how the development time of flies under protein restriction influences lifespan, pre-adult survivorship, and pupation height; thus, to correlate the interrelationship between pre-adult and adult traits.

### Shortening of pupation and pigmentation time

The results of the pupation and pigmentation study suggest that a restricted protein diet influences the time taken by larvae to pupate and pigment. The results also show that the flies respond to protein restriction according to their nutritional needs and are able to sustain the effect of lowest protein concentration by increasing their pupation and pigmentation time (Fig. 1A,B, Table 2). Further, not many reports have explored the pre-adult stage-specific effect of diet on the overall pre-adult development of the flies. Hence, our study reports that the pace of action of DR on each concomitant stage of development is almost consistent and hence does not vary drastically (except for DR10%; Fig. 1C) across different stages of fruit fly development.

### Differential egg-to-adult development duration under varied yeast levels

Diet restriction is capable of modulating development time in *D. melanogaster* (Güler et al., 2015). The main focus of this study is that the development time alterations from protein restriction are highly variable (Table 2), suggesting that there is a differential pressure of protein restriction on the nutritional requirements of the flies. In this case, development time can be prolonged due to unsatisfied differential protein requirements, and flies with a low concentration of proteins in their diet show increased development time (DR10%), while DR30% and DR50% showed faster development due to their nutritional requirement and satiety (Fig. 2A,B). On the other hand, protein concentrations of 20% and 40% in diet are sufficient to maintain the development time of the flies that is similar to AL (Fig. 2A). Further, DR30% and DR50% show that the females with DR develop faster compared to males. This is not uncommon, however, it is important to note that regarding other DRs, faster female development is not evident; particularly at DR10% and DR20%, probably due to the protein constraint. This is unavoidable for the larvae and hence the female larvae have to wait and extend their developmental duration so as to feed sufficiently and satisfy their energy reservoir. Therefore, it will be interesting to find out the mechanisms of such significant sex-based differences and diet×sex interactions. In addition to this, our results add evidence to the existing negative correlation between development time and protein concentration, and are in line with the studies reported elsewhere (Güler et al., 2015; Reis, 2016; Stefana et al., 2017), but counter the results of Kashyap and Shakarad (2016), who stated that high protein diet lengthens development time, which might be because of their use of direct casein for high protein diet preparation rather than yeast itself, which was previously used for their standard media preparation.

### Pre-adult survivorship remains unaffected under different DR

The pre-adult survivorship of flies remained unchanged in different tested protein concentrations (Fig. 3A), suggesting that the diet imposed does not cause any detrimental effect during pre-adult stage of fruit flies. This might be because the flies prefer to increase their development time, thereby ensuring their nutrient satiety, which in turn leads to little or no effect on pre-adult survivorship upon DR. The absence of such effect of restricted diet on pre-adult survivorship also shows that diet does not necessarily influence the successful survival of the pre-adults, but does in turn influence long term fitness traits beneficially. Further, studies have reported that maternal nutrition influences the nutrient environment of their offspring, wherein a rich-diet-fed offspring did not show any potential difference in survivorship when derived from a rich-diet-fed mother (Prasad et al., 2003). However, the mRNA transcripts mediating survival show plasticity upon inheritance to offspring from flies fed on a poor diet (Crofton et al., 2018). Hence, countering these aspects, the effect of diet on pre-adult survivorship was assayed; thereby avoiding the possibility of maternal effects on the offspring, and the results of our study show unaltered pre-adult survivorship upon various protein restricted diets.

### Larval feeding rate under different DRs is variable

The larval feeding rate under different diets is seen to vary, and thereby indicates that diet protein levels can influence larval feeding rate. Additionally, it will be interesting to study the exact mechanisms and address why the lowest feeding rate is observed at DR10% (Fig. 3B). Previous studies have shown a positive correlation between development time and feeding rate in fruit fly populations selected for faster development (Burnet et al., 1977; Borash et al., 2000). Further, studies have shown that larvae with a higher feeding rate tend to accumulate higher lipid reserves as compared to their counterparts (Borash and Ho, 2001; Foley and Luckinbill, 2001). Taken together with reference to the higher feeding rate at DR30%, DR40% and DR50%, larvae can be expected to possess higher lipid reserves as compared to that of DR10%. Surprisingly, the trend followed by pupation time and larval feeding rate across different diets is very similar, suggesting that the larvae feed faster and pupate faster, thereby validating our own results.

### Pupation height under different DR

The results of the pupation height assay reveal that DR10% showed the lowest pupation height (Fig. 4A) and this could be due to the reduced energy usage for pupation height. However, this is debatable because DR10% showed increased development time and lower pupation height, contrary to the suggestion that the flies could lower pupation height to shorten developmental duration (Prasad et al., 2001; Paranjpe et al., 2004). This also stands contrary to the study conducted by Buck et al. (2000), thereby showing that the normally existing positive correlation between development time and pupation height is challenged upon DR implementation (Fig. 4B). Further, since pupation height can influence the chances of a fly's survival (Casares and Carracedo, 1987), reducing protein concentration by nearly half of the AL food (DR50%) enables pupation height similar to that of AL, thereby probably ensuring successful eclosion and survival. Therefore, it will be interesting to study how an excess of protein in the AL diet (as compared to the results of DR50%) causes reduced pupation height (Fig. 4A) and whether this excess 50% protein is influential enough to change the phenotype of rover larvae to sitter larvae or vice versa (de Belle and Sokolowski, 1987, 1989; Sokolowski et al., 1997). Hence, the evolution of polymorphism in larval foraging behavior and energy allocation for pupation site can be taken to suggest that development time and pupation height might play some role in mediating lifespan. Now, the inter-relationship between diet restriction, pupation height and energy investment trade-off between development time and pupation can be further clearly justified. Therefore, our results reveal that DR is capable of increasing the pupation height of the organism, and can also possibly improve overall fitness under the imposed nutrition-limited environment.

### Delayed development increases adult lifespan under low protein diet

The effect of variable protein restriction on fruit flies, irrespective of their sex, shows lifespan extension at low protein concentrations of DR10% and DR20% as compared to that of AL, wherein the results of DR10% causing lifespan extension can probably be explained due to the positive correlation that exists between development time and lifespan (Figs 2A and 5C). In the case of DR20% (an intermediate concentration between the increased development time causing DR10% and decreased development time as seen in DR30%), the increased lifespan observed in flies can be explained due to the long term exposure of this diet (pre-adult development+adult lifespan stages together), which is capable of influencing lifespan significantly, but not the development time alone (as it comprised a short term exposure of DR 20% during pre-adult stage only). The results are consistent with previous studies regarding DR effect on lifespan (Stefana et al., 2017; Klepsatel et al., 2018), and on development time (Klepsatel et al., 2018). This positive correlation is also in line with a previous study that showed that faster pre-adult development resulted in a shorter lifespan (Yadav and Sharma, 2014). Our results suggesting that lifespan extension upon variations in protein concentrations alone is in contrast to the studies of Bruce et al. (2013), thereby showing that interplay between carbohydrate and protein ratios need not essentially explain the results of lifespan modifications upon DR. Further, these results can be taken to suggest that increased yeast concentration will impose negative effects on lifespan. Increased development time upon protein reduction can positively influence lifespan, indicating that the reduced larval diet does not necessarily intrude in extending lifespan upon protein restriction. Although the results of the current study are not sufficient to draw any conclusion based on the evolutionary significance of DR proteins on the studied traits (as it is observed in a single generation), it would not be surprising to expect a cumulative effect of DR protein in the regulation of such life-history traits across at least 20–30 generations. This will enable us to understand the genetic and evolutionary importance of DR paradigm and to study how the evolutionary trajectories of life-history traits change across generations, even though very few studies have managed to study them in *D. melanogaster*.

### Conclusion

The results of our study show that the effect of protein restriction on fruit flies is significantly different with respect to the traits such as pupation time, pigmentation time, pupation height and development time, while is not different with respect to pre-adult survivorship and lifespan. It also reveals that reduced protein concentrations are capable of increasing pupation height as compared to the control, thereby possibly enabling a chance of better eclosion and survival of the flies. Thus, the present study involved the use of same flies to assess all the traits, and hence we think that our results provide a multi-dimensional approach of the influence of DR on fruit flies. Therefore, our results along with other studies on DR, development time and lifespan can be taken to suggest a positive correlation between these two traits, alongside testing the existing positively and negatively reported correlations among different traits.

## MATERIALS AND METHODS

### Fly culture and maintenance

A population of wild-type strains of fruit flies *D. melanogaster* (*Canton* S-CS) was shared by Prof. Vijay Kumar Sharma (Chronobiology Lab, JNCASR, Bangalore) and was maintained at constant temperature (25±0.5°C; mean±s.d.) and humidity (70±5%) on a 21-day discrete generation cycle. This population comprising ∼1200 adult flies (roughly equal number of males and females) was maintained in a plexiglass cage (25 cm×20 cm×15 cm) supplemented with standard banana-jaggery medium. In the current study, flies were fed with five different concentrations of protein restricted diet (DR10%, 20%, 30%, 40% and 50%) and AL in glass vials (9 cm height×2.4 cm diameter) under temperature of ∼25°C (±0.5°C), humidity (∼70%), light intensity (∼600 lux) and light/dark 12:12 h (LD12:12 h) cycles during the experiment. Agar (HIMEDIA) and dry yeast Gloripan instant in food media were used for this study.

### Development time assay

Prior to the egg collection for development time assay, in order to avoid unknown development time differences due to possible variations in egg retention in the female body, flies were presented with a cut plate of AL corn medium for 1 h, which was replaced with another fresh cut plate of AL corn medium for the next 2 h. Eggs laid over the 2 h window were collected for the assay and half of this egg-collection window, i.e. first 1 h was considered as the start (zero-0 h) of pre-adult development time assay. Exactly 30 eggs each were dispensed into 9 cm vials containing ∼6 ml food of AL (control-100% protein or 40 gm yeast per l of corn food) and DR (experimental protein or yeast level ranging from 10–50% of that present in AL control; i.e. 4–20 g per l; Table 1), with a total of 30 vials (five vials×six diets) and the above stated dietary regimes. To examine pupation time, we recorded the development of pupae in the interval of 2 h for all the six experimental diet setups. After pupation, we followed the same procedure for pigmentation and adult emergence time assay. The raw data obtained for all these assays were analyzed by taking the average across vials.

**Table 1.**
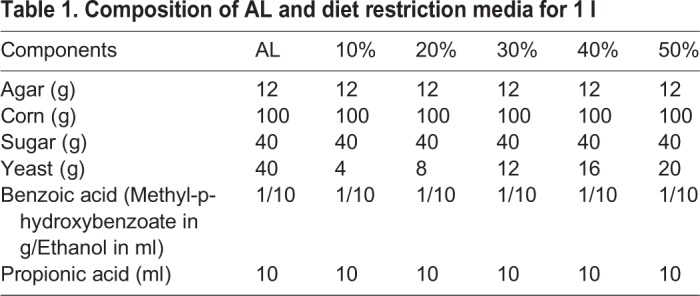
**Composition of AL and diet restriction media for 1 l**

**Table 2.**
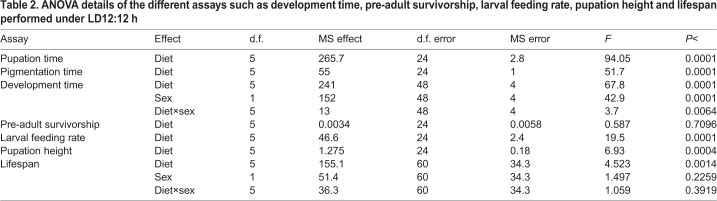
**ANOVA details of the different assays such as development time, pre-adult survivorship, larval feeding rate, pupation height and lifespan performed under LD12:12 h**

### Pre-adult survivorship assay

Similar to the protocol mentioned previously, eggs laid over the 2 h window were collected and exactly 30 eggs each were dispensed into 9 cm vials containing different diets, with a total of 30 vials (five vials×six diets). The pre-adult survivorship was assessed by dividing the total number of flies eclosed from each vial at the end of the experiment by the total number of eggs dispensed in each vial, i.e. 30.

### Larval feeding rate assay

Eggs laid over the 2 h window were collected and approximately 30 eggs were dispensed into 9 cm vials containing different diets, with a total of 30 vials (five vials×six diets). The larval feeding rate was counted as the number of sclerite retractions per minute of the early third instar larvae. A total of ten randomly chosen larvae were counted for their feeding rate in the vial.

### Pupation height assay

Five replicate vials were used, each with 30 eggs for each diet during the assay period. The pupation height was measured after eclosion to avoid any possible external disturbances on eclosion time that might occur during pupation height measurement due to temperature fluctuations or vial handling. Once the flies had eclosed, the pupation height was measured as the distance from the food surface to the center of the empty pupa. In instances of any pupa touching the surface of the medium, the pupation height was marked as zero (Mueller and Sweet, 1986; Joshi and Mueller, 1996).

### Lifespan assay

In order to study the impact of DR alterations upon the genetic correlations prevalent between pre-adult development time and adult lifespan (via avoiding the variations in factors such as egg density, temperature, humidity, etc.), flies emerged from the pre-adult development time assay setup were employed for the lifespan assay at 25°C temperature and ∼70% relative humidity under LD12:12 h. Freshly emerged flies were separated under light phase of LD12:12 h after being anesthetized using a mild amount of CO_2_. The whole assay involved imposing diet/protein restriction from the egg stage, in the respective diet based food (ranging from 10%, 20%, 30%, 40%, and 50% of available yeast) and the unaltered AL control food. The setup consisted of AL and DR food (with protein concentrations ranging from 10–50%) each containing a group of ten unmated males and females dispensed into each separate vial containing 6 ml of corn food. The vials were checked for death of flies every day and the surviving flies were transferred gently into fresh food vials every fourth day, this practice was continued throughout the lifespan of flies till the death of last fly in each vial during which the temperature and humidity were found to be constant throughout the assays. The lifespan of a fly was calculated as the number of days it survived post-emergence.

### Statistical analyses

Data from development time assay was subjected to main effects analysis of variance (ANOVA); treating development time as dependent factor, while diet and sex being independent factors. Similarly, pupation height and pre-adult survivorship data were also subjected to ANOVA, with diet as an independent factor. Lifespan data was also subjected to main effects ANOVA, treating diet and sex as independent factors. All statistical analyses were done using ANOVA, followed by Tukey's HSD test for multiple comparisons on STATISTICA for Windows Release 7 (StatSoft Inc. 1995, 2004). The correlation between development time versus pupation height was done using Sigmaplot, Systat Software.

## References

[BIO042952C1] Aggarwal D. D., Ranga P., Kalra B., Parkash R., Rashkovetsky E. and Bantis L. E. (2013). Rapid effects of humidity acclimation on stress resistance in *Drosophila melanogaster*. *Comp. Biochem. Physiol. A Mol. Integr. Physiol.* 166, 81-90. 10.1016/j.cbpa.2013.05.012 23688505

[BIO042952C2] Borash D. J. and Ho G. T. (2001). Patterns of selection: stress resistance and energy storage in density-dependent populations of Drosophila melanogaster. *J. Insect Physiol.* 47, 1349-1356. 10.1016/S0022-1910(01)00108-1 12770141

[BIO042952C3] Borash D. J., Te'otonio H., Rose M. R. and Mueller L. D. (2000). Density-dependent natural selection in *Drosophila*: correlations between feeding rate, development time and viability. *J. Evol. Biol.* 13, 181-187. 10.1046/j.1420-9101.2000.00167.x

[BIO042952C4] Bruce K. D., Hoxha S., Carvalho G. B., Yamada R., Wang H.-D., Karayan P., He S., Brummel T., Kapahi P. and Ja W. W. (2013). High carbohydrate-low protein consumption maximizes *Drosophila* lifespan. *Exp. Gerontol.* 48, 1129-1135. 10.1016/j.exger.2013.02.003 23403040 PMC3687007

[BIO042952C5] Buck S., Vettraino J., Force A. G. and Arking R. (2000). Extended longevity in *Drosophila* is consistently associated with a decrease in developmental viability. *J. Gerontol. A Biol. Sci. Med. Sci.* 55, B292-B301. 10.1093/gerona/55.6.B292 10843346

[BIO042952C6] Burnet B., Sewell D. and Bos M. (1977). Genetic analysis of larval feeding behaviour in *Drosophila melanogaster*. II. Growth relations and competition between selected lines. *Genet. Res.* 30, 149-161. 10.1017/S0016672300017559 4217753

[BIO042952C7] Casares P. and Carracedo M. C. (1987). Pupation height in *Drosophila*: sex differences and influence of larval developmental time. *Behav. Genet.* 17, 523-535. 10.1007/BF01073119 3426507

[BIO042952C8] Chiang H. C. and Hodson A. C. (1950). An analytical study of population growth in *Drosophila melanogaster**.* *Ecol. Monogr.* 20, 173-206. 10.2307/1948580

[BIO042952C9] Chippindale A. K., Alipaz J. A., Chen H. W. and Rose M. R. (1997). Experimental evolution of accelerated development in *Drosophila*. 1. Developmental speed and larval survival. *Evolution.* 51, 1536-1551. 10.1111/j.1558-5646.1997.tb01477.x 28568633

[BIO042952C10] Crofton A. E., Cartwright E. L., Feitzinger A. A. and Lott S. E. (2018). Effect of Larval Nutrition on Maternal mRNA Contribution to the *Drosophila* Egg. *G3 (Bethesda).* 8, 1933-1941. 10.1534/g3.118.200283 29666195 PMC5982822

[BIO042952C11] de Belle J. S. and Sokolowski M. B. (1987). Heredity of rover/sitter: alternative foraging strategies of *Drosophila melanogaster* larvae. *Heredity* 59, 73-83. 10.1038/hdy.1987.98

[BIO042952C12] de Belle J. S. and Sokolowski M. B. (1989). Rover/sitter foraging behavior in *Drosophila melanogaster*: genetic localization to chromosome2L using compound autosomes. *J. Insect Behav.* 2, 291-299. 10.1007/BF01068056

[BIO042952C13] Foley P. A. and Luckinbill L. S. (2001). The effects of selection for larval behavior on adult life-history features in Drosophila melanogaster. *Evolution* 55, 2493-2502. 10.1111/j.0014-3820.2001.tb00764.x 11831665

[BIO042952C14] Grandison R. C., Piper M. D. W. and Partridge L. (2009). Amino-acid imbalance explains extension of lifespan by dietary restriction in *Drosophila*. *Nature.* 462, 1061-1064. 10.1038/nature08619 19956092 PMC2798000

[BIO042952C15] Güler P., Ayhan N., Koşukcu C. and Önder B. S. (2015). The effects of larval diet restriction on developmental time, preadult survival, and wing length in *Drosophila melanogaster*. *Turk. J. Zool.* 39, 395-403. 10.3906/zoo-1305-42

[BIO042952C16] Hoffmann A. A., Hallas R., Anderson A. R. and Telonis-Scott M. (2005). Evidence for a robust sex-specific trade-off between cold resistance and starvation resistance in *Drosophila melanogaster*. *J. Evol. Biol.* 18, 804-810. 10.1111/j.1420-9101.2004.00871.x 16033551

[BIO042952C17] Joshi A. and Mueller L. D. (1993). Directional and stabilizing density-dependent natural selection for pupation height in *Drosophila melanogaster*. *Evolution.* 47, 176-184. 10.1111/j.1558-5646.1993.tb01208.x 28568099

[BIO042952C18] Joshi A. and Mueller L. D. (1996). Density-dependent natural selection in *Drosophila*: trade-offs between larval food acquisition and utilization. *Evol. Ecol.* 10, 463 10.1007/BF01237879

[BIO042952C19] Kapahi P., Kaeberlein M. and Hansen M. (2017). Dietary restriction and lifespan: lessons from invertebrate models. *Ageing. Res. Rev.* 39, 3-14. 10.1016/j.arr.2016.12.005 28007498 PMC5476520

[BIO042952C20] Kashyap K. and Shakarad M. N. (2016). Effect of high protein diet during larval growth on adult physiology in *Drosophila Melanogaster*. *Int. J. Adv. Sci. Eng. Technol.* 4, 203-208.

[BIO042952C21] Klepsatel P., Procházka E. and Gáliková M. (2018). Crowding of *Drosophila* larvae affects lifespan and other life-history traits via reduced availability of dietary yeast. *Exp. Gerontol.* 110, 298-308. 10.1016/j.exger.2018.06.016 29932967

[BIO042952C22] Lee K. P. (2015). Dietary protein: carbohydrate balance is a critical modulator of lifespan and reproduction in *Drosophila melanogaster*: a test using a chemically defined diet. *J. Insect. Physiol.* 75, 12-19. 10.1016/j.jinsphys.2015.02.007 25728576

[BIO042952C23] Lee K. P., Simpson S. J., Clissold F. J., Brooks R., Ballard J. W. O., Taylor P. W., Soran N. and Raubenheimer D. (2008). Lifespan and reproduction in *Drosophila*: New insights from nutritional geometry. *Proc. Natl. Acad. Sci. USA* 105, 2498-2503. 10.1073/pnas.0710787105 18268352 PMC2268165

[BIO042952C24] Lihoreau M., Poissonnier L.-A., Isabel G. and Dussutour A. (2016). *Drosophila* females trade off good nutrition with high-quality oviposition sites when choosing foods. *J. Exp. Biol.* 219, 2514-2524. 10.1242/jeb.142257 27284071

[BIO042952C25] Mair W., Goymer P., Pletcher S. D. and Partridge L. (2003). Demography of dietary restriction and death in *Drosophila*. *Science* 301, 1731-1733. 10.1126/science.1086016 14500985

[BIO042952C26] Mair W., Piper M. D. W. and Partridge L. (2005). Calories do not explain extension of life span by dietary restriction in *Drosophila*. *PLoS Biol.* 3, e223 10.1371/journal.pbio.0030223 16000018 PMC1140680

[BIO042952C27] Metaxakis A. and Partridge L. (2013). Dietary restriction extends lifespan in wild-derived populations of *Drosophila melanogaster*. *PLoS ONE* 8, e74681 10.1371/journal.pone.0074681 24040317 PMC3769260

[BIO042952C28] Mueller L. D. and Sweet V. F. (1986). Density-dependent natural selection in *Drosophila*: evolution of pupation height. *Evolution* 40, 1354-1356. 10.1111/j.1558-5646.1986.tb05761.x 28563511

[BIO042952C29] Nunney L. and Cheung W. (1997). The effect of temperature on body size and fecundity in female *Drosophila melanogaster*: evidence for adaptive plasticity. *Evolution.* 51, 1529-1535. 10.1111/j.1558-5646.1997.tb01476.x 28568642

[BIO042952C30] Ormerod K. G., LePine O. K., Abbineni P. S., Bridgeman J. M., Coorssen J. R., Mercier A. J. and Tattersall G. J. (2017). *Drosophila* development, physiology, behavior, and lifespan are influenced by altered dietary composition. *Fly (Austin)* 11, 153-170. 10.1080/19336934.2017.1304331 28277941 PMC5552271

[BIO042952C31] Paranjpe D. A., Anitha D., Sharma V. K. and Joshi A. (2004). Circadian clocks and life-history related traits: is pupation height affected by circadian organization in *Drosophila melanogaster*? *J. Genet.* 83, 73-77. 10.1007/BF02715831 15240911

[BIO042952C32] Prasad N. G., Shakarad M., Anitha D., Rajamani M. and Joshi A. (2001). Correlated responses to selection for faster development and early reproduction in *Drosophila*: the evolution of larval traits. *Evolution* 55, 1363-1372. 10.1111/j.0014-3820.2001.tb00658.x 11525460

[BIO042952C33] Prasad N. G., Shakarad M., Rajamani M. and Joshi A. (2003). Interaction between the effects of maternal and larval levels of nutrition on pre-adult survival in *Drosophila melanogaster*. *Evol. Eco. Res.* 5, 903-911.

[BIO042952C34] Reis T. (2016). Effects of synthetic diets enriched in specific nutrients on *drosophila* development, body fat, and lifespan. *PLoS ONE* 11, e0146758 10.1371/journal.pone.0146758 26741692 PMC4704830

[BIO042952C35] Rodrigues M. A., Martins N. E., Balancé L. F., Broom L. N., Dias A. J. S., Fernandes A. S. D., Rodrigues F., Sucena É. and Mirth C. K. (2015). *Drosophila melanogaster* larvae make nutritional choices that minimize developmental time. *J. Insect Physiol.* 81, 69-80. 10.1016/j.jinsphys.2015.07.002 26149766

[BIO042952C36] Rogina B. and Helfand S. L. (2004). Sir2 mediates longevity in the fly through a pathway related to calorie restriction. *Proc. Natl. Acad. Sci. USA* 101, 15998-16003. 10.1073/pnas.0404184101 15520384 PMC528752

[BIO042952C37] Sameoto D. D. and Miller R. S. (1968). Selection of pupation site by *Drosophila melanogaster* and *D. simulans*. *Ecology* 49, 177-180. 10.2307/1933580

[BIO042952C38] Silva-Soares N. F., Nogueira-Alves A., Beldade P. and Mirth C. K. (2017). Adaptation to new nutritional environments: larval performance, foraging decisions, and adult oviposition choices in *Drosophila suzukii*. *BMC Ecol.* 17, 21 10.1186/s12898-017-0131-2 28592264 PMC5463304

[BIO042952C39] Simpson S. J., Sibly R. M., Lee K. P., Behmer S. T. and Raubenheimer D. (2004). Optimal foraging when regulating intake of multiple nutrients. *Anim. Behav.* 68, 1299-1311. 10.1016/j.anbehav.2004.03.003

[BIO042952C40] Sokal R. R., Ehrlich P. R., Hunter P. E. and Schlager G. (1960). Some factors affecting pupation site of *Drosophila*. *Ann. Entomol. Soc. Am.* 53, 174-182. 10.1093/aesa/53.2.174

[BIO042952C41] Sokolowski M. B., Pereira H. S. and Hughes K. (1997). Evolution of foraging behavior in *Drosophila* by density-dependent selection. *Proc. Natl. Acad. Sci. USA* 94, 7373-7377. 10.1073/pnas.94.14.7373 9207098 PMC23828

[BIO042952C42] Stefana M. I., Driscoll P. C., Obata F. and Pengelly A. R. (2017). Developmental diet regulates *Drosophila* lifespan via lipid autotoxins. *Nat. Commun.* 8, 1384 10.1038/s41467-017-01740-9 29123106 PMC5680271

[BIO042952C43] Tu M.-P. and Tatar M. (2003). Juvenile diet restriction and the aging and reproduction of adult *Drosophila melanogaster*. *Aging Cell.* 2, 327-333. 10.1046/j.1474-9728.2003.00064.x 14677635

[BIO042952C44] Vandal N. B. and Shivanna N. (2007). Temperature dependent larval pupation site preference in different species of *Drosophila*. *Povolzhasky J. Ecol.* 2C, 91-105.

[BIO042952C45] Yadav P. and Sharma V. K. (2014). Correlated changes in life history traits in response to selection for faster pre-adult development in the fruit fly *Drosophila melanogaster**.* *J. Exp. Biol.* 217, 580-589. 10.1242/jeb.093864 24523501

[BIO042952C46] Yang C. H., Belawat P., Hafen E., Jan L. Y. and Jan Y. N. (2008). *Drosophila* egg-laying site selection as a system to study simple decision-making processes. *Science* 319, 1679-1683. 10.1126/science.1151842 18356529 PMC2581776

